# Chronic Intermittent Hypoxia Exerts CNS Region-Specific Effects on Rat Microglial Inflammatory and TLR4 Gene Expression

**DOI:** 10.1371/journal.pone.0081584

**Published:** 2013-12-04

**Authors:** Stephanie M. C. Smith, Scott A. Friedle, Jyoti J. Watters

**Affiliations:** Department of Comparative Biosciences, University of Wisconsin, Madison, Wisconsin, United States of America; Virginia Commonwealth University, United States of America

## Abstract

Intermittent hypoxia (IH) during sleep is a hallmark of sleep apnea, causing significant neuronal apoptosis, and cognitive and behavioral deficits in CNS regions underlying memory processing and executive functions. IH-induced neuroinflammation is thought to contribute to cognitive deficits after IH. In the present studies, we tested the hypothesis that IH would differentially induce inflammatory factor gene expression in microglia in a CNS region-dependent manner, and that the effects of IH would differ temporally. To test this hypothesis, adult rats were exposed to intermittent hypoxia (2 min intervals of 10.5% O_2_) for 8 hours/day during their respective sleep cycles for 1, 3 or 14 days. Cortex, medulla and spinal cord tissues were dissected, microglia were immunomagnetically isolated and mRNA levels of the inflammatory genes iNOS, COX-2, TNFα, IL-1β and IL-6 and the innate immune receptor TLR4 were compared to levels in normoxia. Inflammatory gene expression was also assessed in tissue homogenates (containing all CNS cells). We found that microglia from different CNS regions responded to IH differently. Cortical microglia had longer lasting inflammatory gene expression whereas spinal microglial gene expression was rapid and transient. We also observed that inflammatory gene expression in microglia frequently differed from that in tissue homogenates from the same region, indicating that cells other than microglia also contribute to IH-induced neuroinflammation. Lastly, microglial TLR4 mRNA levels were strongly upregulated by IH in a region- and time-dependent manner, and the increase in TLR4 expression appeared to coincide with timing of peak inflammatory gene expression, suggesting that TLR4 may play a role in IH-induced neuroinflammation. Together, these data indicate that microglial-specific neuroinflammation may play distinct roles in the effects of intermittent hypoxia in different CNS regions.

## Introduction

Intermittent hypoxia (IH) during sleep is a hallmark feature of sleep apnea. IH induces significant cognitive and behavioral deficits that involve disruptions of connections among CNS regions underlying memory processing and executive function. For example, IH promotes apoptosis of hippocampal CA1 and frontal cortical neurons [1], [2] as well as cerebellar Purkinje and fastigial neurons [3] in rodents. Similar gray matter losses have also been observed in hippocampal and para-hippocampal brain regions [4], [5], and the temporal gyrus and cerebellum [6] in humans with sleep apnea. Interestingly, there also appears to be CNS region-specific neuronal vulnerability to IH. Neurons in the hippocampal CA3 region do not undergo apoptosis as a result of IH, whereas CA1 neurons are highly sensitive [1], [2]. To the best of our knowledge, there are no reports of neuronal loss occurring in the brainstem or spinal cord of adult animals exposed to IH protocols. Indeed, the dorsocaudal brainstem and the CA3 regions are considered to be protected from IH-induced neuronal apoptosis [7].

Many mechanisms are thought to contribute to neuronal death following chronic exposure to IH including oxidative stress [8], [9] and inflammation [10], [11]. In particular, enzymatic inhibition and/or genetic deletion of NADPH oxidase [9], [12], [13], [14], cyclooxygenase-2 [10], and inducible nitric oxide synthase [11] protect against IH-induced neuronal apoptosis and cognitive impairment, suggesting that inflammatory processes play an important role in IH-induced neuropathology. Neuronal apoptosis in the CA1 region and cortex (as assessed by single stranded DNA immunostaining) indicates that apoptosis peaks between 1 and 2 days of IH, is significantly decreased by 7 days, and has returned to baseline levels by 14 days [1]. This profile coincides with inducible nitric oxide synthase (iNOS) and cyclooxygenase-2 (COX-2) gene expression in cortical homogenates where mRNA and protein levels peak at 1 day of IH and then decline thereafter [10], [11]. Although COX-2 levels remain elevated above baseline at 14 days of IH [10], iNOS levels remain elevated above basal only for 3 to 7 days [11], suggesting differential gene regulation of these inflammatory molecules in cortical homogenates.

Whether inflammatory molecules besides iNOS and COX-2 are produced in the CNS during IH is not known, nor is the cellular source of these molecules, although iNOS has been attributed to neurons [11]. Microglia, CNS resident immune cells are considered to be contributors to IH-induced neuroinflammation [7], but their activities have never directly been tested in this model of IH [15], [16]. Thus, a major goal of the present studies was to determine the contributions of microglia to IH-induced neuroinflammation over time, and to elucidate the expression profile of inflammatory factor genes. Microglia can be major sources of iNOS and COX-2 in the CNS in other models of CNS neuroinflammation [17], [18], [19], and Toll-like receptor 4 (TLR4) activation is involved in microglial inflammatory responses to many insults [20], [21], [22], [23]. TLR4 is a key pattern recognition receptor whose prototypical ligand is gram-negative lipopolysaccharide (LPS). In addition to iNOS and COX-2, TLR4 activation also leads to the production and release of cytokines including interleukin (IL)-1β, IL-6 and tumor necrosis factor (TNF) α [20], [21], [24]. All of these molecules have been implicated in neuronal injury, death and/or functional impairments in the CNS [10], [25], [26], [27], [28], [29], [30], [31], [32].

Importantly, it is now well established that TLR4 also mediates “sterile inflammation” in the CNS, primarily by responding to a host of endogenous ligands including, proteins associated with cell damage (e.g. heat shock proteins 60 and 70), extracellular matrix turnover, and oxidatively modified lipids [33]. A proteomic study comparing the profile of IH-induced proteins in sensitive (CA1) and resistant (CA3) hippocampal regions showed that several heat shock proteins were upregulated by IH, and some differentially in CA1 versus CA3 [34], suggesting that IH may region-specifically increase endogenous ligands for TLR4 that could contribute to microglial activation and IH-induced neuroinflammation in this region. Interestingly, serum levels of the TLR4 ligands myeloid related proteins (MRP) 8 and 14 [35] correlate with severity of disease in children with OSA [36], suggesting a relationship between IH-induced inflammation and TLR4 activity. No studies to our knowledge have evaluated the levels of TLR4 in the CNS, although recently, upregulated TLR4 levels have been reported on circulating monocytes in adults with obstructive sleep apnea [37].

Because there is CNS regional susceptibility to neuronal damage during chronic IH, in the present studies, we tested the hypothesis that microglia differentially induce inflammatory factor gene expression in response to IH, and that the expression profiles of these genes will differ over time in IH-sensitive and -resistant CNS regions. Adult rats were exposed to a paradigm of intermittent hypoxia (2 min intervals of 10.5% O2) for 8 hours/day during their respective sleep cycles for 1, 3 or 14 days. Cortex, medulla and spinal cord tissues were dissected, microglia were immunomagnetically isolated and mRNA levels of inflammatory genes were compared to those in tissue homogenates containing all CNS cells. We found that the inflammatory genes induced by IH differed in microglia from different CNS regions, and that the general temporal profiles of inflammatory gene expression varied in IH-sensitive and -resistant CNS regions. Further, we observed that the expression of inflammatory genes in microglia versus regional tissue homogenates frequently differed, especially at the 14 day timepoint, indicating that cells other than microglia also produce inflammatory factors in response to chronic IH exposure. Lastly, microglial TLR4 mRNA levels were strongly upregulated in a region- and time-dependent manner, and its expression often coincided with increased inflammatory gene expression, suggesting that TLR4 may play a role in IH-induced neuroinflammation.

## Materials and Methods

### Animals

#### Ethics Statement

This study was carried out in strict accordance with the recommendations in the Guide for the Care and Use of Laboratory Animals of the National Institutes of Health. All surgical and experimental procedures were approved by the University of Wisconsin Madison Institutional Animal Care and Use Committee. All efforts were made to minimize the number of animals used and their suffering.

Experiments were performed on Sprague-Dawley rats weighing 150 g±20 g (Harlan, Indianapolis, IN). All animals were maintained in an AAALAC-accredited animal facility and housed under standard conditions, with a 12 hour light/dark cycle with food and water available *ad libitum*.

### Reagents

Neural Tissue Dissociation Kit, anti-PE magnetic beads, and MS columns were purchased from Miltenyi Biotech (Germany). PE-mouse anti-rat CD11b was purchased from BD Biosciences (San Jose, CA). Hank's Buffered Salt Solution (HBSS) was purchased from Cellgro (Herndon, VA). TRI reagent was purchased from Sigma Aldrich (St. Louis, MO). Glycoblue reagent was purchased from Ambion (Austin, TX). MMLV Reverse transcriptase was purchased from Invitrogen (Carlsbad, CA). Oligo dT, Random Primers, and RNAse inhibitor were purchased from Promega (Madison, WI). Primers were designed using Primer 3 software and were purchased from Integrated DNA Technologies (Coralville, IA). Power SYBR green was purchased from Applied Biosystems (Foster City, CA).

### Intermittent Hypoxia (IH) Exposures

Animals were placed in computer controlled, custom-manufactured chambers that mix O_2_, N_2_, and CO_2_ to the desired concentration with a flow rate of 4 L/min to enable rapid dynamics and minimal CO_2_ accumulation. The treatment groups were exposed to alternating 2 minute episodes of hypoxia (10.5% inspired O2) and normoxia (21% inspired O_2_) for 8 h/day during their respective night cycle for 1, 3, or 14 days. The control groups (normoxia for 1, 3 or 14 days) underwent identical handling, but their chambers were continually flushed with room air (21% inspired O_2_). Following daily exposure, rats were returned to their cages. Normoxic and corresponding IH groups were harvested at the same time. Because there was no significant difference between normoxic groups in the expression of any of the genes evaluated at any time point, we combined all normoxic groups for subsequent statistical analyses and graphical representations.

### CD11b^+^ Cell Isolation

Rats were harvested 16 hours after their last hypoxic exposure, and CD11b^+^ cells were isolated using previously described methods [38], [39]. Briefly, rats were euthanized and perfused with cold phosphate buffered saline (PBS). The cortex, medulla (pontomedullary junction to obex) and spinal cord (C1-L6) were removed and dissociated into a single cell suspension using the neural tissue dissociation kit (Miltenyi) according to the manufacturer's protocol. Myelin was removed by high speed centrifugation at 850xg in a 0.9 M solution of sucrose in HBSS. CD11b^+^ cells were tagged with a PE-conjugated anti-CD11b^+^ antibody followed by an anti-PE antibody conjugated to a magnetic bead. Magnetically-tagged CD11b^+^ cells were isolated using MS columns according to the Miltenyi MACS protocol. Prior to CD11b^+^ cell isolation an aliquot of tissue cell suspension was taken for CNS homogenate tissue analysis. Reagents were kept chilled at 4°C and cells were kept on ice whenever possible. As we have previously shown, this method results in >97% pure population of CD11b^+^/CD45 ^low^ cells [38], [39]. Isolated CD11b^+^ cells will subsequently be referred to as “microglia.”

### RNA extraction/reverse transcription

RNA was extracted from tissue homogenates and freshly-isolated microglia according to the TriReagent protocol, with the addition of Glycoblue during the isopropanol incubation. cDNA was synthesized from 1 µg of total RNA using MMLV Reverse Transcriptase as previously described [38].

### Quantitative PCR

cDNA was used in real-time quantitative PCR with Power SYBR Green using either the ABI StepOne or ABI 7500 Fast system. Primers (Table 1) were designed using Primer 3 software and the specificity was assessed using NCBI BLAST. Primer efficiency was tested through the use of serial dilutions. Verification that the dissociation curve had a single peak with an observed Tm consistent with the amplicon length was performed for every PCR reaction. C_T_ values from duplicate measurements were averaged and normalized to levels of the ribosomal RNA, 18s. Relative gene expression was determined using the relative standard curve method as previously described [38].

**Table 1:** 

Gene	Forward Primer (5′)	Reverse Primer (5′)
18s	AACGAGACTCTCGG ATGCTAA	CCGGACATCTAAGGGCATCA
Cyclooxygenase 2 (COX-2)	TGTTCCAACCCATGTCAAAA	CGTAGAATCCAGTCCGGGTA
Inducible nitric oxide synthetase (iNOS)	AGGGAGTGTTGTTCCAGGTG	TCTGCAGGATGTCTTGAACG
Tumor necrosis factor α (TNFα)	TCCATGGCCCAGACCCTCACAC	TCCGCTTGGTTTGCTACG
Interleukin 1β (IL1β)	CTGCAGATGCAATGGAAAGA	TTGCTTCCAAGGCAGACTTT
Interleukin-6 (IL-6)	GTGGCTAAGGACCAAGACCA	GGTTTGCCGAGTAGACCTCA
TLR4	AGGCAGCAGGTGGAATTGTATC	TCGAGGCTTTTCCATCCAATAG

### Statistical analysis

Statistical analyses were performed on the normalized, interpolated C_T_ values from the standard curves for each gene, as previously described [40], using a one-way ANOVA followed by the Holm-Sidak multiple comparisons post hoc test using Sigma Plot 11.0 software (San Jose, CA). Data sets that failed normality were logarithmically transformed prior to statistical analyses. Statistical significance was set at the 95% confidence limit (p<0.05). A single symbol above a bar represents p<0.05; two symbols p<0.01; and three symbols p<0.001. Quantitative data are expressed as the mean ±1 SEM of up to 3 independent experiments containing n = 4–6 animals/group for normoxia and IH treatments.

## Results

### IH time-dependently increases the expression of inflammatory genes (but not TNFα) in cortical microglia

**Figure 1:**
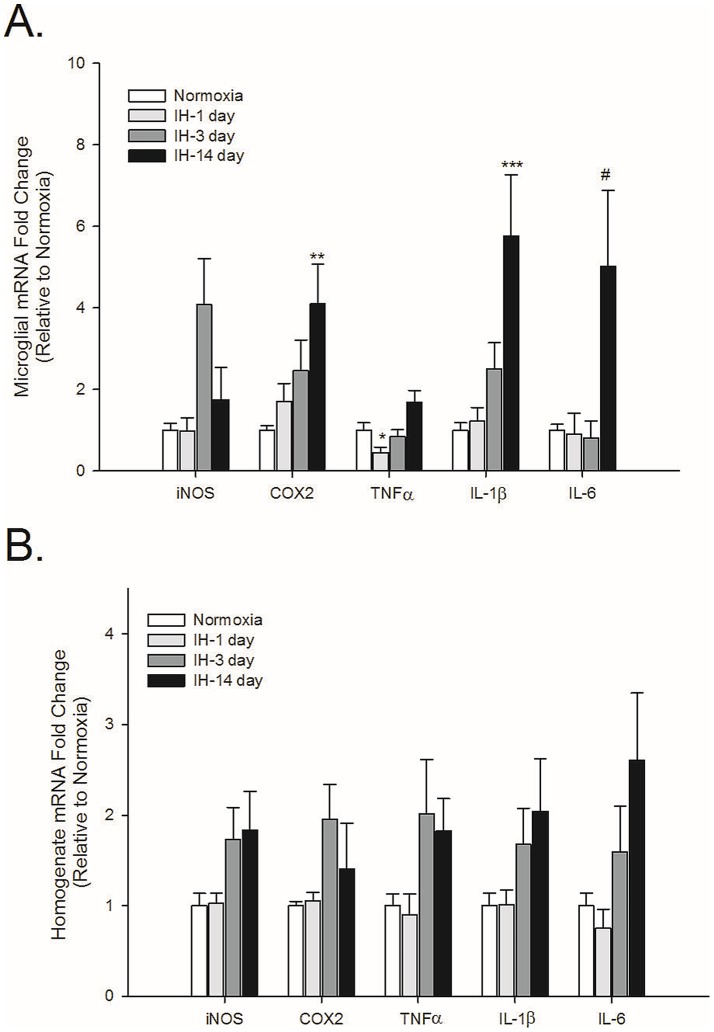
Intermittent hypoxia-induced inflammatory gene expression peaks in cortical microglia at 14 days of exposure. iNOS, COX-2, TNFα, IL-1β and IL-6 gene expression was analyzed by qRT-PCR in immunomagnetically-separated microglia (A) or tissue homogenates (B) from the cortex of healthy male rats exposed either to normoxia or IH for 1, 3 or 14 days. Means +/− 1 SEM are presented relative to expression in normoxic controls. *p<0.05; **p<0.01; ***p<0.001; ^#^p = 0.09.

IH treatment increased cortical microglial inflammatory gene expression (compared to normoxic controls) over the course of the 14-day IH exposure (Fig. 1A). There was a statistically significant 4-fold increase in COX-2 (p = 0.002) and a 6-fold increase in IL-1β (p = <0.001) mRNA levels after 14 days of IH. Although there was an apparent 5-fold increase in the expression of IL-6 after 14 days (p = 0.09) and a 4-fold increase in iNOS expression at 3 days (p = 0.074), these genes did not reach statistical significance by ANOVA. Interestingly, relative to expression in normoxia, TNFα mRNA levels significantly decreased at 1 day (p = 0.024) but had increased back to baseline levels by 3 days. In contrast, in cortical tissue homogenates (Fig. 1B), although the mRNA levels of all genes showed an increase of approximately 2- fold after 3 days of IH and this increase was maintained at 14 days, they did not reach statistical significance as determined by ANOVA although TNFα came close (p = 0.055; iNOS p = 0.09; COX-2 p = 0.299; IL-1β p = 0.25; IL-6 p = 0.11).

### IH promotes early and long-lasting increases in IL-1β and IL-6 gene expression in medullary microglia, but multiple inflammatory genes are upregulated in homogenates

**Figure 2:**
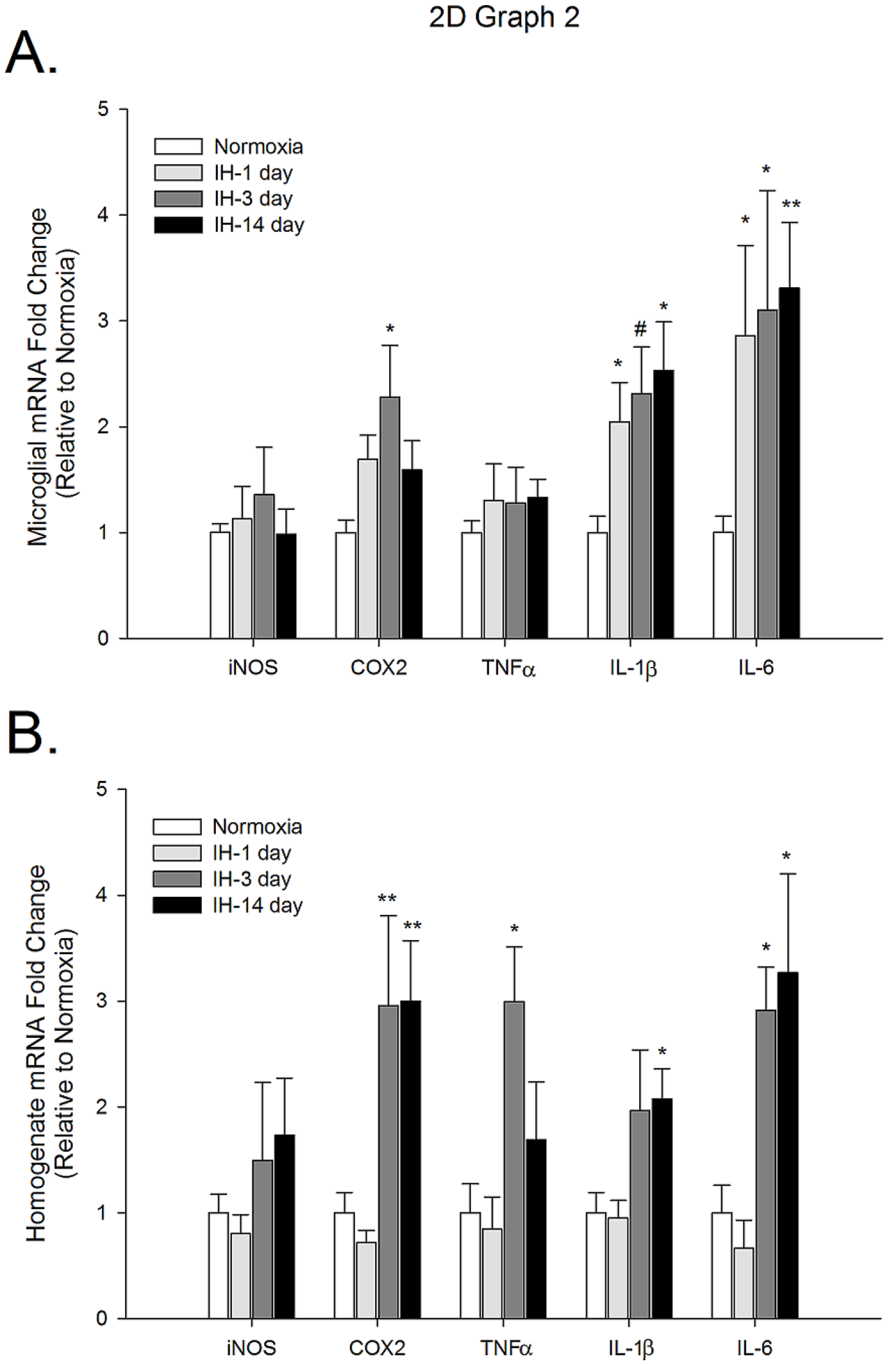
Intermittent hypoxia-induced inflammatory gene expression differs temporally in brainstem microglia and brainstem tissue homogenates. iNOS, COX-2, TNFα, IL-1α and IL-6 gene expression was analyzed by qRT-PCR in immunomagnetically-separated microglia (A) or tissue homogenates (B) from the brainstem of healthy male rats exposed either to normoxia or IH for 1, 3 or 14 days. Means +/− 1 SEM are presented relative to expression in normoxic controls. *p<0.05; **p<0.01; ^#^p = 0.06.

Medullary microglia appeared to have a more rapid inflammatory response to IH relative to cortical microglia. Whereas in cortical microglia IL-1β and IL-6 mRNA levels did not appear to peak until 14 days of IH, in the medulla, microglial IL-1β and IL-6 mRNA levels were increased at 1 day of IH and remained elevated at 14 days (Fig. 2A). Interestingly, microglial IL-1β and IL-6 expression profiles paralleled each other in both the medulla and cortex. In addition, while cortical microglial COX-2 mRNA levels were highest at 14 days, in the medulla, microglial COX-2 was elevated at 3 days of IH and seemed to be returning to baseline levels by 14 days. IH exposure did not change iNOS (p = 0.56) or TNFα (p = 0.71) mRNA levels at any timepoint tested here. Unlike microglia, medullary homogenates behaved similarly to cortical homogenates (Fig. 2B). COX-2, TNFα, IL-1β and IL-6 mRNA levels were significantly increased (∼2–3 fold) at 3 days of IH, and with the exception of TNFα, the expression of these genes remained elevated at 14 days. iNOS levels in medullary homogenates remained unchanged over the course of IH treatment (p = 0.479).

### IH promotes transient inflammatory gene expression in spinal microglia but longer lasting effects in homogenates

**Figure 3:**
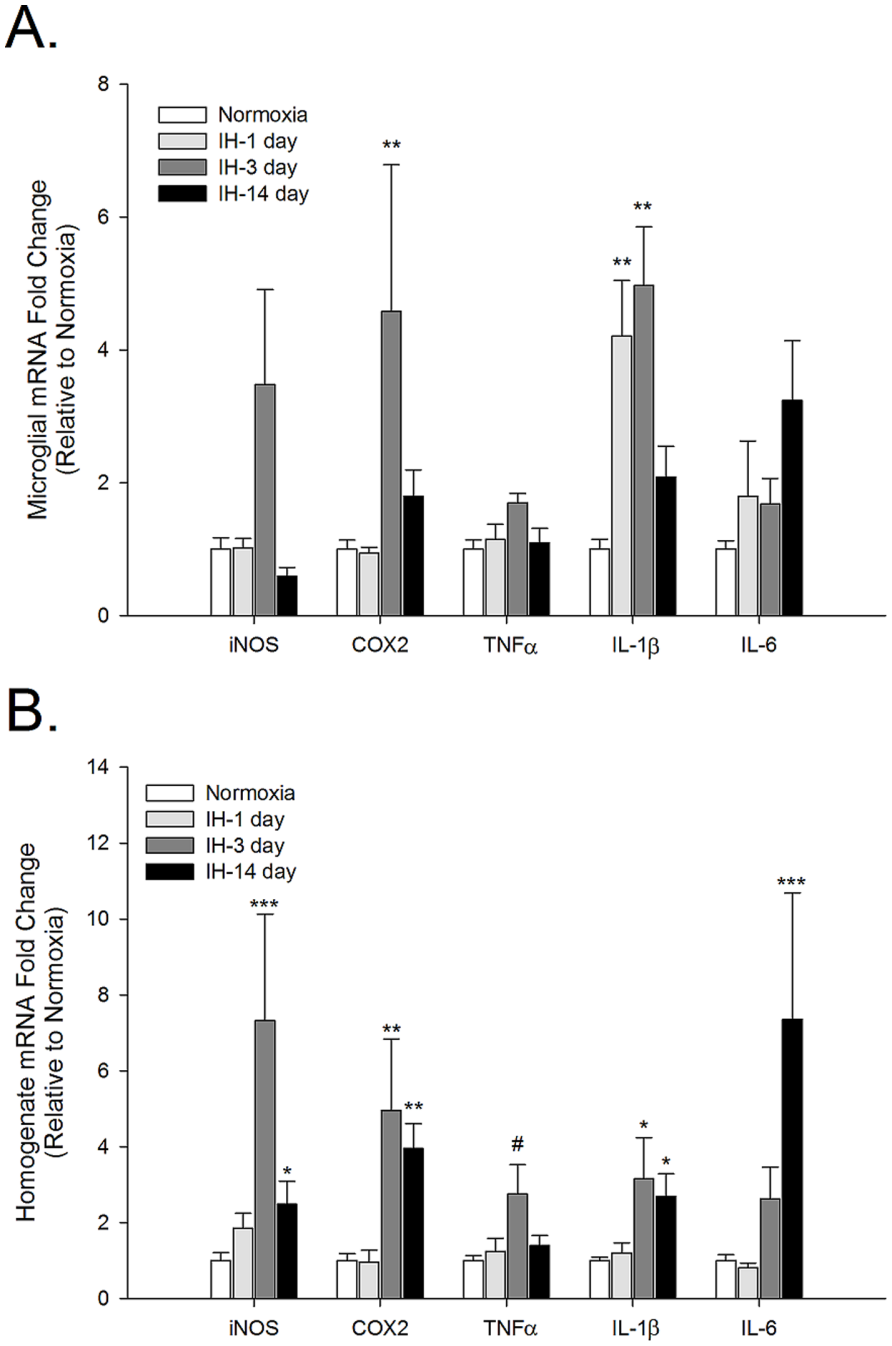
Intermittent hypoxia-induced inflammatory gene expression is rapid and transient in spinal microglia but sustained in spinal tissue homogenates. iNOS, COX-2, TNFα, IL-1β and IL-6 gene expression was analyzed by qRT-PCR in immunomagnetically-separated microglia (A) or tissue homogenates (B) from the spinal cord of healthy male rats exposed either to normoxia or IH for 1, 3 or 14 days. Means +/− 1 SEM are presented relative to expression in normoxic controls. *p<0.05; **p<0.01; ***p<0.001; ^#^p = 0.074.

IL-1β was the only inflammatory gene to exhibit a strong upregulation within 1 day of IH in spinal microglia (p = <0.001) (Fig. 3A). However, by 3 days of IH, iNOS, COX-2, IL-1β and IL-6 mRNA levels were increased by 3–5 fold, but only COX-2 (p = 0.007) and IL-1β (p<0.001) levels attained statistical significance (iNOS p =  0.12; TNFα p =  0.28; IL-6 p =  0.099). COX-2 and IL-1β expression returned to basal levels by 14 days of IH exposure. IH did not appear to increase microglial IL-6 mRNA levels until 14 days although this 3-fold change was not statistically significant (p = 0.099). Spinal homogenates behaved very similarly to brainstem homogenates where the expression of all inflammatory genes examined was increased between 3 and 8-fold at 3 days of IH (Fig. 3B). COX-2 and IL-1β expression remained elevated at 14 days of IH (p = 0.003 and p = 0.033 respectively), but iNOS mRNA levels were returning to baseline despite remaining significantly elevated above basal levels (p = 0.044). Also, as in medullary homogenates, spinal homogenate IL-6 mRNA levels were highest at 14 days of IH (p = <0.001) while TNFα mRNA levels appeared to peak at 3 days (though not statistically significantly; p = 0.074).

### IH differentially increases microglial TLR4 expression in cortex, medulla and spinal cord

**Figure 4:**
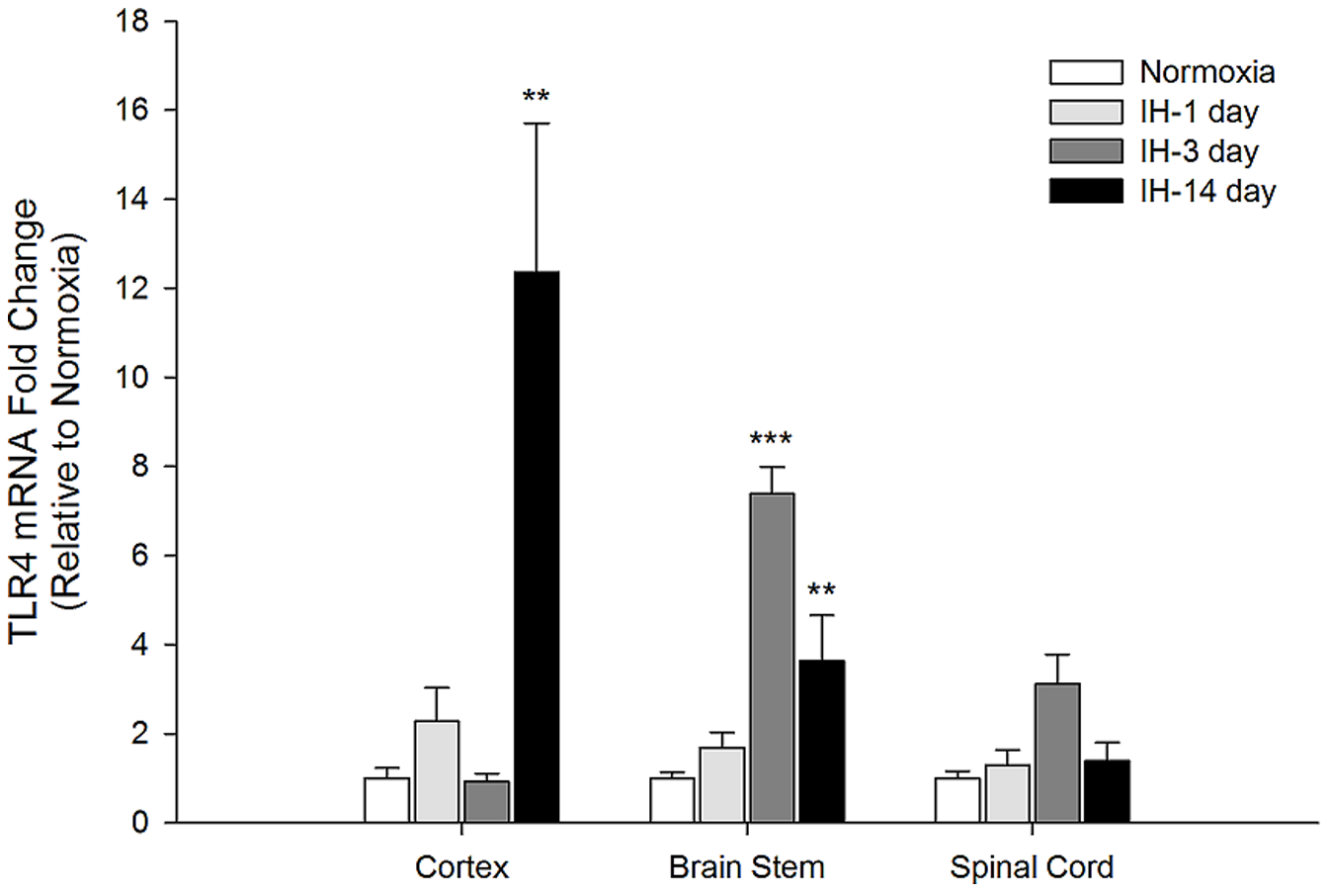
Intermittent hypoxia-induced TLR4 gene expression in microglia differs by time and CNS region. TLR4 gene expression was analyzed by qRT-PCR in immunomagnetically-separated microglia from the cortex, brainstem and spinal cord of healthy male rats exposed either to normoxia or IH for 1, 3 or 14 days. Means +/− 1 SEM are presented relative to expression in normoxic controls. **p<0.01; ***p<0.001; ^#^p = 0.085.

Because TLR4 plays an important role in mediating inflammatory gene induction in microglia, and endogenous TLR4 ligands are increased in IH-susceptible CNS regions, we evaluated the expression of TLR4 over time following IH exposure (Fig. 4). Very interestingly, the largest increase in TLR4 mRNA levels occurred in microglia from the cortex where expression was increased by approximately 12-fold at 14 days of IH (p = 0.001). The apparent 2-fold increase at 1 day of IH was not significant (p = 0.398 by ANOVA). TLR4 expression in microglia from the medulla was increased by 7-fold at 3 days of IH (p = <0.001), and remained elevated (by 4-fold at 14 days (p = 0.001). In spinal microglia, TLR4 expression was not significantly increased at any time point tested, although an approximate 3-fold increase at 3 days of IH was observed (p =  0.085 by ANOVA).

## Discussion

In this study we were interested in understanding whether microglial inflammatory gene expression was altered by IH, whether those responses differed based on CNS region from which the microglia were derived, if there was temporal regulation of IH-induced inflammatory gene expression, and whether assessments of neuroinflammation in tissue homogenates were accurately reflective of microglial activities in specific. We show here that IH induces differential inflammatory gene profiles in microglia from different CNS regions, and that patterns of IH-induced inflammatory gene expression differ in tissue homogenates (in which all cell types are present) versus isolated microglia from the same region. Lastly, we find that the expression of the pattern recognition receptor TLR4 is time- and CNS region-dependently upregulated by IH in microglia in a manner that coincides with microglial inflammatory gene expression. This report is the first to directly evaluate microglial phenotype following intermittent hypoxia exposure, and to reveal differences in microglial responses based on CNS region and time of exposure. Our data also indicate that microglia are likely not the only CNS cell type contributing to neuroinflammation following chronic exposure to IH.

Microglia make up approximately 5-10% of all CNS cells, with the cortex having more microglia than caudal CNS regions such as the brainstem and spinal cord (unpublished observations; Nikodemova et al. manuscript in revision). Because microglia comprise such a small percentage of total CNS cells, changes in microglial gene expression may not necessarily be evident in tissue homogenates unless the changes are very strong. For example, the approximately 50% reduction in TNFα or the 4-fold increase in COX-2 mRNA levels in cortical microglia are not apparent in cortical homogenates. Our data also indicate that cell types other than microglia can and do synthesize inflammatory molecules in response to IH exposure. For example, in spinal tissue homogenates, the mRNA levels of iNOS, COX-2 and IL-6 are significantly elevated above normoxic levels at 14 days of IH when expression of these genes in microglia is not different from controls. The identity of the cells contributing to the expression of these neuroinflammatory mediators is not yet known, but such results suggest that conclusions made about microglial activities based on the expression/presence of inflammatory molecules in tissue homogenates may not always be accurate.

The observation that microglia respond to the same IH stimulus differently in different parts of the CNS may be related to differences in the local CNS environment as well as potential fundamental differences in the microglia themselves. In the cortex, microglial inflammatory gene expression (COX-2, IL-1β and IL-6) appears to increase over time after IH. However, these effects are gene-specific because iNOS expression had returned to baseline by 14 days and TNFα expression was not increased at any time point evaluated. Indeed, TNFα expression was significantly decreased in cortical microglia at 1 day of IH. This was the only CNS region in which we observed a decrease in the expression of any inflammatory gene. We do not yet know the significance of this rapid and acute TNFα downregulation, as the biological functions of microglial produced TNFα versus TNFα produced by other cell types have not been distinguished. Additional studies are necessary to delineate mechanisms underlying this microglial-specific inhibition of TNFα gene expression in the cortex.

When comparing the cortex where IH-induced neuronal apoptosis occurs [1], [34], to the spinal cord where there are no reports of IH-induced neuronal loss, we find that microglia from these two CNS regions behave very differently from each other. In cortical microglia, we observe a general pattern of time-dependent increases in inflammatory gene expression that appear to peak at 14 days of IH. Spinal microglia on the other hand, respond acutely and transiently to the IH stimulus such that the expression of inflammatory genes had returned to basal levels by 14 days. Although we do not yet know the reasons underlying these regional differences, we hypothesize that the availability of endogenous TLR4 ligands may play a role, at least in part. Endogenous TLR4 ligands are increased in the IH-susceptible CA1 hippocampal region, but not in the CA3 region where the neurons appear to be resistant to apoptotic effects of IH [34], [41]. Whereas similar proteomic studies have not been performed in the cortex, because there is significant IH-induced cortical neuron loss, we suspect that cortical results would be similar to those in the CA1 region. Why certain CNS regions appear to be susceptible to or protected from IH-induced neuronal loss is not yet clear, but differential induction of IH-regulated proteins [41] and changes in basal metabolism in different regions [34] have been suggested. Regardless, we hypothesize that these differences contribute to region-specific alterations in microglial inflammatory gene expression profiles and temporal dynamics.

To the best of our knowledge, there are no reports of neuronal loss occurring in the dorsocaudal brainstem of adult animals exposed to IH protocols [7], [42], although some brainstem regions including the nucleus tractus solitarius (nTS) and the nucleus ambiguus in the ventrolateral medulla are susceptible in the developing CNS [43]. However, in the dorsocaudal brainstem, IH increases NMDA receptor subunit expression [44] and alters antioxidant responses in pontine neurons [45]. Thus, although the dorsocaudal brainstem is protected from IH-induced neuronal apoptosis, neuronal function in several brainstem nuclei are altered by IH. Our observations that inflammatory gene expression in isolated microglia and homogenates from the medulla had some similarities to both the cortex and spinal cord are consistent with the varied activities of IH in different brainstem nuclei. The changes in microglial inflammatory and TLR4 gene expression in our medullary samples may therefore reflect responses of heterogeneous microglial populations that cannot be distinguished using the present immunomagnetic isolation methodology.

TLR4 expression and activity is increased on monocytes from patients with sleep apnea [37], and ligands for TLR4 are increased in the serum of children with sleep apnea [36]. TLR4 is also upregulated in the CNS in many injuries and neurodegenerative disease processes [24], [46], [47], [48], [49]. Although TLR4 mRNA and protein levels are increased by chronic sustained hypoxia in microglia *in vitro* [50], whether this also occurs following exposure to intermittent patterns of hypoxia, *in vivo*, was not known prior to this study. We found that TLR4 gene expression was very strongly upregulated in cortical microglia after 14 days of IH and in medullary microglia at 3 and 14 days, consistent with the timing of peak inflammatory gene expression in the respective CNS regions. Since the function of upregulated TLR4 in CNS disease is thought to promote and/or maintain neuroinflammation, chronically upregulated microglial TLR4 in the cortex may contribute to IH-induced neuroinflammation and neuronal loss. *In vitro*, sustained hypoxia transcriptionally upregulates TLR4 expression via transactivation of the TLR4 promoter by hypoxia inducible factor (HIF)1α in macrophages [51]. Interestingly, we also observe a strong increase in HIF-1α mRNA at 14 days of IH in microglia (data not shown), supporting the idea that IH may contribute to chronically elevated microglial TLR4 levels via similar HIF-1-dependent transcriptional mechanisms.

The biological significance of microglia-mediated neuroinflammation in response to IH may be different in different CNS regions, especially given the differences in time domains. For example, in the cortex, microglial inflammatory gene expression appears to be maximal at 14 days (the longest time point measured here), suggesting that microglia may contribute to neuroinflammation and neuronal loss/damage in the chronic state. In contrast, spinal microglia also respond to IH by increasing inflammatory gene expression, but these effects appear to be rapid and acute as inflammatory mRNA levels had returned to baseline by 14 days. Although the function of transient microglial inflammation in the spinal cord is not yet known, our recent studies have suggested that acute spinal inflammation, contributed to in part by microglia, can abrogate phrenic nerve long-term facilitation (pLTF), a form of spinal motor neuron plasticity [52]. Interestingly, in rats exposed to IH for 7 days, pLTF is greatly enhanced compared to normoxia-exposed rats [53], suggesting that this form of motor neuron plasticity may return when IH-induced microglial inflammation subsides.

The IH model used here simulates a hallmark feature of sleep apnea. Recent estimates indicate that up to 34% of men and 13% of women between 30 and 70 yrs of age are affected by obstructive sleep apnea [54] which causes serious neural morbidities including neuroinflammation, neuronal death and cognitive impairment [7], [55]. However, equally as important, sleep disordered breathing and associated IH is also severe in >50% of patients with other major health problems including ischemic (e.g. stroke), traumatic (e.g. spinal injury), neurodegenerative (e.g. Alzheimer's, Parkinson's, ALS, MS) and genetic neural disorders (e.g. Down's syndrome, Fragile X) [56], [57], [58], [59], [60], [61], [62]._ENREF_27 IH triggers the upregulation of endogenous TLR4 ligands in the CNS [34], and we find that TLR4 expression is upregulated in microglia by IH. Because TLR4 activation causes neurodegeneration [46], and several neurodegenerative diseases (e.g. AD, ALS and MS) are associated with abnormal TLR4 function [24], [47], [48], [49], collectively, our data suggest that IH-induced TLR4 upregulation may play a previously unrecognized role in many CNS disorders. Thus, IH-induced neuroinflammation and neuronal death may accelerate or exacerbate ongoing pathology associated with other primary disorders. We suggest that IH-induced microglial activation may therefore be a critical, underappreciated contributor to many seemingly unrelated neural disorders.
